# Priority-Lasso: a simple hierarchical approach to the prediction of clinical outcome using multi-omics data

**DOI:** 10.1186/s12859-018-2344-6

**Published:** 2018-09-12

**Authors:** Simon Klau, Vindi Jurinovic, Roman Hornung, Tobias Herold, Anne-Laure Boulesteix

**Affiliations:** 1 Institute for Medical Information Processing, Biometry and Epidemiology, University of Munich, Munich, Germany; 2 Department of Internal Medicine III, University of Munich, Munich, Germany

**Keywords:** Cox regression, Lasso, Multi-omics data, Penalized regression, Prediction model, Priority-lasso

## Abstract

**Background:**

The inclusion of high-dimensional omics data in prediction models has become a well-studied topic in the last decades. Although most of these methods do not account for possibly different types of variables in the set of covariates available in the same dataset, there are many such scenarios where the variables can be structured in blocks of different types, e.g., clinical, transcriptomic, and methylation data. To date, there exist a few computationally intensive approaches that make use of block structures of this kind.

**Results:**

In this paper we present priority-Lasso, an intuitive and practical analysis strategy for building prediction models based on Lasso that takes such block structures into account. It requires the definition of a priority order of blocks of data. Lasso models are calculated successively for every block and the fitted values of every step are included as an offset in the fit of the next step. We apply priority-Lasso in different settings on an acute myeloid leukemia (AML) dataset consisting of clinical variables, cytogenetics, gene mutations and expression variables, and compare its performance on an independent validation dataset to the performance of standard Lasso models.

**Conclusion:**

The results show that priority-Lasso is able to keep pace with Lasso in terms of prediction accuracy. Variables of blocks with higher priorities are favored over variables of blocks with lower priority, which results in easily usable and transportable models for clinical practice.

**Electronic supplementary material:**

The online version of this article (10.1186/s12859-018-2344-6) contains supplementary material, which is available to authorized users.

## Background

Many cancers are heterogeneous diseases regarding biology, treatment response and outcome. For example, in the context of acute myeloid leukemia (AML), a variety of classifiers and recommendations were published to guide treatment decisions [1]. We and others have recently shown that gene expression markers as well as mutational profiling are able to improve risk prediction based on standard clinical markers [2–5]. Other types of biomarkers such as copy number variation data or methylation data may also be used for this purpose in the future. However, irrespective of the considered specific end point (e.g., overall survival, resistant disease, early death) no model is currently able to precisely predict the outcome of AML patients. To date, the most powerful prognostic models are based on cytogenetics and gene expression markers [6].

In the present paper, we use the term *omics* to denote molecular biomarkers measured through high-throughput experiments. Beyond the example of AML mentioned above, the integration of multiple types of omics biomarkers with the aim of improved prediction accuracy has been a focus of much attention in the past years, see for example [7] and references therein. While prediction modelling using a single type of omics markers is a well-studied topic, it is not clear how different types of biomarkers should be handled simultaneously when deriving a prediction model.

In addition to the highly important topic of prediction accuracy, encompassing both discrimination ability and calibration, clinical reality requires analysts to take aspects related to *usability* into account when developing prediction models for clinical practice. Firstly, a model including several hundreds/thousands of variables is much more difficult to implement in clinical practice than a model including only a handful of variables. *Sparsity* is thus an important aspect of the model which contributes to its practical utility in clinical settings. Secondly, a model including variables that are already included in routine diagnostics — such as genetic alterations as recommended by the European LeukemiaNet (ELN) in the case of AML [1], or variables that can be easily assessed such as age or common clinical variables — are more likely to be accepted by physicians than a model including variables measured with new and/or expensive technologies, maybe even at the expense of a slightly lower prediction accuracy. These two points are arguments in favor of models that (preferably) include a small number of variables selected from particular “favorite” sets of variables — as opposed to, say, a large number of variables selected from genome-wide data.

Another aspect related to practical usability is the *transportability* of a prediction model, i.e. the possibility for potential users to apply the prediction model to their own data based on information provided by the model developers [8]. Penalized regression methods yielding sparse models typically yield better transportable models than black-box machine learning algorithms [8, 9]. For example, to apply a Lasso logistic regression model [10] for making predictions for their own patients, users only need the fitted regression coefficients and names of the selected variables to compute the score and, if they want to compute predicted probabilities, the fitted intercept. In contrast, a prediction tool constructed using, for example, the random forest algorithm, can be applied by other researchers or clinicians only if they have access to a software object (such as the output of the R function ‘randomForest’ if the package of the same name is used) or the dataset and the code used to construct it — which may become obsolete after a few years. In this sense, Lasso logistic regression is preferable to random forest as far as transportability and sustainability are concerned. Note that model interpretation is also particularly easy with sparse penalized regression methods.

Finally, coming back to prediction accuracy, we note that medical experts often have some kind of prior knowledge regarding the information content of different sets of variables. For example, they often expect (a particular set of) the clinical variables to have high prediction ability and a large proportion of the gene expression variables to be less relevant. Such prior knowledge should ideally be taken into account while constructing a prediction model.

Motivated by the need, in the context of AML research and other fields, for sparse transportable models selecting preferably variables that are easy to collect or expected to yield good prediction accuracy, we suggest *priority-Lasso*, a simple Lasso-based approach. Priority-Lasso is a hierarchical regression method which builds prediction rules for patient outcomes (e.g., a time-to-event, a response status or a continuous outcome) from different blocks of variables including high-throughput molecular data while taking clinicians’ preference into account. More precisely, clinicians define “blocks” of variables (which may simply correspond to the type of data, e.g., the block of methylation variables or the block of gene expression variables) and order these blocks according to their level of priority. The prediction model is then fitted in a stepwise manner: In turn, each block of variables is considered as a covariate matrix in Lasso regression, in the sequence of priority specified by the clinician; see the “Methods” section for more details.

The priority-Lasso procedure is fast and simple. It can cope with all the types of outcome variables accepted by Lasso and, more generally, inherits its properties. The hierarchical principle of priority-Lasso can essentially also be applied to extensions of Lasso, including but not limited to elastic net [11], adaptive Lasso [12] or stability selection [13], but also, more generally, to other prediction methods applicable to high-dimensional covariate data. Last but not least, note that the priority sequence imposed by the clinician merely determines which blocks are prioritized over other blocks with respect to rendering predictive information that is contained in several blocks. Predictive information of blocks with low priority that is not contained in blocks with high priority is still exploited by priority-Lasso (see “Principles of priority-Lasso” section for details).

The rest of this paper is structured as follows. Section “Methods” presents the priority-Lasso method and its implementation in detail. In “Results” section, the method is illustrated with different settings through an application to AML data and compared to standard Lasso in terms of accuracy and included variables. The considered outcome is the survival time and the considered types of data are comprised of clinical data, the mutation status of several genes and gene expression data. Most importantly, prediction models are fitted on a training dataset and subsequently validated on an independent dataset following the recommendations by Royston and Altman [14].

## Methods

We first provide a non-technical introduction into the principles of priority-Lasso in “Principles of priority-Lasso” section to make these concepts accessible to readers without strong statistical background and to give a succinct overview. We present the method formally in “Formalization of priority-Lasso” section, treat its implementation in “R package prioritylasso” section, and describe in “Validation” section the validation strategy inspired from Royston and Altman [14] adopted in our illustrative example.

### Principles of priority-Lasso

Priority-Lasso is a method that can construct a prediction model for a clinical outcome of interest (e.g., a time to event or a response status and continuous outcome) based on candidate variables, using an available training dataset. Before running priority-Lasso, the user is required to first specify a block structure for the covariates where each covariate belongs to exactly one of *M* blocks and, second, a priority order of these blocks.

A block may be of a particular data type, for example “clinical data”, “gene expression data” or “methylation data”, but the classification of variables into blocks may also be finer. For example, clinical data may be divided into two blocks, e.g., the demographic data (e.g., age or sex) in a first block and clinical data related to the tumor in the second block. Once the blocks of variables are defined, the clinician orders them according to their level of priority. High priority should be given to blocks which are easy and/or inexpensive to collect or are already routinely collected in clinical practice.

After this definition, the prediction model is fitted in a stepwise manner. In the first step, a Lasso model is fitted to the block with highest priority. The goal of this step is simply to explain the largest possible part of the variability in the outcome variable by the covariates from the block with highest priority. In the second step, a Lasso model is fitted to the block with second highest priority using the linear score from the first step as an *offset*, i.e., this linear score is forced into the model with coefficient fixed to 1. In the special case of a metric outcome, this corresponds to fitting a second Lasso model (without the offset) to the residuals from the first Lasso model using the block with second highest priority as covariate matrix. The goal of this second step is thus to use the variables from the second block to explain remaining variability in the outcome variable that could not be explained by covariates from the first block.

In the third step, a Lasso regression is fitted to the block with third highest priority using the linear score from the second step as offset. The special case of a metric outcome is correspondingly equivalent to fitting a Lasso model to the residuals from the second Lasso model using the block with third highest priority. This procedure is iterated until all blocks have been considered in turn. Thus, in the case of a metric outcome, at each step the current block is fitted to the residuals of the previous step. Generalizing to other types of outcome variables, in each step the current block is fitted to the outcome conditional on all blocks with higher priority that were considered in the previous steps. In this way, blocks of variables with low priority enter the model only if they explain variability that is not explainable by blocks with higher priority. Compared to non-hierarchical approaches, priority-Lasso tends to yield models in which variables from the most prioritized blocks play a more important role.

This procedure was motivated by the fact that there is frequently a strong overlap of predictive information across the considered blocks. For example, some gene expression and gene mutation variables can be associated with the same phenotype, which is why these two different types of omics data may contain similar predictive information. Moreover, clinical covariates and omics covariates often carry similar predictive information. If, in priority-Lasso, a block A is given a higher priority than a block B, this means that the part of the predictive information contained in A and B that is common to both blocks will be obtained from block A. The larger the number of blocks, the lower the information contained in individual blocks, that is not contained in any other block. Thus, in the presence of a large number of blocks there is a high chance that priority-Lasso will exclude variables from blocks of low priority, because the predictive information contained therein may also be contained in the data of blocks of higher priority. Therefore, by providing a priority sequence, the analyst can decide which blocks should be prioritized over others with respect to providing predictive information redundant among blocks. The chosen priority sequence can, however, be expected to have a limited impact on the prediction error for the following reason: If a block A with strong predictive power is attributed a low priority, its predictive power will nevertheless be exploited in the prediction rule. This is because the proportion of the variability of the outcome variable that is only explainable by block A will still be unexplained before block A is considered as a covariate block in the iterative procedure.

### Formalization of priority-Lasso

In the following description, we consider *M* blocks of continuous or binary variables that are all to be penalized, and a continuous outcome variable for the sake of simplicity. Extensions to time-to-event and binary outcomes are straightforward using the corresponding variants of Lasso (Cox Lasso and logistic Lasso, respectively, see [15] and [10, 16]). The extension to multicategorical variables is also straightforward using an appropriate coding of the variables.

Let *x*_*ij*_ denote the observed value of the *j*th variable (*j*=1,…,*p*) for the *i*th subject (*i*=1,…,*n*) and *y*_*i*_ denote the observed outcome of subject *i*. For simplicity it is assumed that each variable is centered to have mean zero over the *n* observations. The standard Lasso method [10] estimates the regression coefficients *β*_1_,…,*β*_*p*_ of the *p* variables by minimizing the expression

$$\sum\limits_{i=1}^{n}\left(y_{i} - \sum\limits_{j=1}^{p}x_{ij}\beta_{j}\right)^{2} + \lambda \sum\limits_{j=1}^{p}|\beta_{j}|$$

with respect to *β*_1_,…,*β*_*p*_, where *λ* is a so-called penalty parameter. This method performs both regularization (shrinkage of the estimates) and variable selection (i.e., some of the estimates are shrunken to zero, meaning that the variable is excluded from the model). The amount of shrinkage is determined by the parameter *λ*, which is considered as a tuning parameter of the method and is in practice most often chosen using cross-validation.

We now adapt our notation to the case of variables forming groups that is considered in this paper. From now on, the observations of the *p*_*m*_ variables from block *m* for subject *i* are denoted as $x_{i1}^{(m)},\ldots,x_{{ip}_{m}}^{(m)}$, for *i*=1,…,*n* and *m*=1,…,*M*. The number of blocks *M* usually ranges from 2 to, say, 10 in practice, while the number *p*_*m*_ of variables often varies strongly across the blocks. For example, blocks of clinical variables typically include a very small number of variables, say, *p*_*m*_≈10, while blocks of molecular variables from high-throughput experiments may include several tens or hundreds of thousands of variables.

Similarly to the definition of $x_{ij}^{(m)}$, $\beta _{j}^{(m)}$ denotes the regression coefficient of the *j*th variable from block *m*, for *j*=1,…,*p*_*m*_, while $\hat {\beta }_{j}^{(m)}$ stands for its estimated counterpart.

Let us further denote as ***π***=(*π*_1_,…,*π*_*M*_) the permutation of (1,…,*M*) that indicates the priority order: *π*_1_ denotes the index of the block with highest priority, while *π*_*M*_ is the index of the block with the lowest priority. For example, if *M*=4, ***π***=(3,1,4,2) means that the third block has highest priority, the first block has second highest priority, and so on. Conversely, the priority level of a given block is indicated by the position of its index in the vector ***π***.

In the first step of priority-Lasso, the variables from block *π*_1_ are used to fit a Lasso regression model. The coefficients $\beta _{1}^{(\pi _{1})},\dots,\beta _{p_{\pi _{1}}}^{(\pi _{1})}$ are estimated by minimizing

$$ \sum\limits_{i=1}^{n} \left(y_{i} - \sum\limits_{j=1}^{p_{\pi_{1}}} x_{ij}^{(\pi_{1})} \beta_{j}^{(\pi_{1})}\right)^{2} + \lambda^{(\pi_{1})} \sum\limits_{j=1}^{p_{\pi_{1}}} \left|\beta_{j}^{(\pi_{1})}\right|. $$

The linear predictor fitted in step 1 is given as

$$\hat{\eta}_{1,i}(\boldsymbol{\pi}) = \hat{\beta}_{1}^{(\pi_{1})}x_{i1}^{(\pi_{1})} + \ldots + \hat{\beta}_{p_{\pi_{1}}}^{(\pi_{1})}x_{{ip}_{\pi_{1}}}^{(\pi_{1})}. $$

In “Principles of priority-Lasso” section we noted that this linear predictor is used as an offset in the second step in which we fit a Lasso model to block *π*_2_. However, the linear score $\hat {\eta }_{1,i}(\boldsymbol {\pi })$ tends to be over-optimistic with respect to the information usable for predicting *y*_*i*_ that is contained in block *π*_1_. The reason for the latter is that *y*_*i*_ was part of the data used for obtaining the estimates $\hat {\beta }_{1}^{(\pi _{1})}, \dots, \hat {\beta }_{p_{\pi _{1}}}^{(\pi _{1})}$, which are then used to calculate $\hat {\eta }_{1,i}(\boldsymbol {\pi })$. This overoptimism is essentially similar to the well-known overoptimism that results from estimating the prediction error of a prediction rule using the observations in the training dataset. When using this over-optimistic estimate $\hat {\eta }_{1,i}(\boldsymbol {\pi })$ as an offset in the second step, the influence of block *π*_2_ conditional on the influence of block *π*_1_ will tend to be underestimated. The reason for this is that by considering the over-optimistic estimate $\hat {\eta }_{1,i}(\boldsymbol {\pi })$ as an offset, a part of the variability in *y*_*i*_ is removed that is actually not explainable by block *π*_1_ but would possibly be explainable by block *π*_2_. As noted above, this problem results from the fact that *y*_*i*_ is contained in the training data used for estimating $\beta _{1}^{(\pi _{1})}, \dots, \beta _{p_{\pi _{1}}}^{(\pi _{1})}$. As a solution to this problem we suggest estimating the offsets *η*_1,*i*_(***π***) using cross-validation in the following way: 1) Split the dataset *S* randomly into *K* approximately equally sized parts *S*_1_,…,*S*_*K*_; 2) For *k*=1,…,*K*: obtain estimates $\hat {\beta }_{S\setminus S_{k}, 1}^{(\pi _{1})}, \dots, \hat {\beta }_{S\setminus S_{k}, p_{\pi _{1}}}^{(\pi _{1})}$ of the Lasso coefficients using the training data *S*∖*S*_*k*_ and for all *i*∈*S*_*k*_ (*k*=1,…,*K*), calculate the cross-validated offsets as

$$\hat{\eta}_{1,i}(\boldsymbol{\pi})_{\operatorname{CV}} = \hat{\beta}_{S\setminus S_{k}, 1}^{(\pi_{1})}x_{i1}^{(\pi_{1})} + \ldots + \hat{\beta}_{S\setminus S_{k}, p_{\pi_{1}}}^{(\pi_{1})}x_{{ip}_{\pi_{1}}}^{(\pi_{1})}. $$

In the second step the coefficients of the variables in block *π*_2_ are thus estimated by minimizing

$${} \sum\limits_{i=1}^{n} \left(y_{i} \,-\, \hat{\eta}_{1,i}(\boldsymbol{\pi})_{\operatorname{CV}} \,-\, \sum\limits_{j=1}^{p_{\pi_{2}}} x_{ij}^{(\pi_{2})} \beta_{j}^{(\pi_{2})}\right)^{2} \,+\, \lambda^{(\pi_{2})} \sum\limits_{j=1}^{p_{\pi_{2}}} \left|\beta_{j}^{(\pi_{2})}\right|. $$

Using $\hat {\eta }_{2,i}(\boldsymbol {\pi }) = \hat {\eta }_{1,i}(\boldsymbol {\pi })_{\operatorname {CV}} + \hat {\beta }_{1}^{(\pi _{2})}x_{i1}^{(\pi _{2})} + \ldots + \hat {\beta }_{p_{\pi _{2}}}^{(\pi _{2})}x_{{ip}_{\pi _{2}}}^{(\pi _{2})}$ as an offset in the third step in which we fit a Lasso model to block *π*_3_ could again lead to underestimating the influence of block *π*_3_ conditional on the influences of blocks *π*_1_ and *π*_2_. This is because, analogously to the first step, the estimates $\hat {\beta }_{1}^{(\pi _{2})}, \dots, \hat {\beta }_{p_{\pi _{2}}}^{(\pi _{2})}$ used to calculate $\hat {\eta }_{2,i}(\boldsymbol {\pi })$ are overly well adapted to the residuals $y_{i} - \hat {\eta }_{1,i}(\boldsymbol {\pi })_{\operatorname {CV}}$. Therefore, we again suggest to calculate cross-validated estimates, $\hat {\eta }_{2,i}(\boldsymbol {\pi })_{\operatorname {CV}}$, of the offsets analogously to the first step.

Priority-Lasso proceeds analogously for the remaining groups until the final (*M*th) fit, where the following linear predictor is obtained:

$$\hat{\eta}_{M,i}(\boldsymbol{\pi}) = \sum\limits_{m=1}^{M} \sum\limits_{j=1}^{p_{\pi_{m}}} \hat{\beta}_{j}^{(\pi_{m})} x_{ij}^{(\pi_{m})}. $$

Note that when the offsets are not estimated by cross-validation but the estimates $\hat {\eta }_{1,i}(\boldsymbol {\pi }),\dots,$$\hat {\eta }_{M-1,i}(\boldsymbol {\pi })$ are used, the effects described above of underestimating the conditional influences of the individual blocks accumulate. Thus, the influences of blocks with higher priority are underestimated to a less stronger degree than are blocks with low priority. This could eventually lead to the exclusion of blocks with lower priority that are valuable for prediction. This is particularly problematic in cases in which low priorities are attributed to blocks with high predictive information. Thus, cross-validated offsets may be used to avoid suboptimal models that may result in cases in which the priority sequence does not attribute high priority to blocks with high predictive power. Note, however, that we are not interested in determining priority sequences that perform optimally from a statistical point of view. Instead, the priority sequence reflects the specific needs of the user, who particularly cares about practicability. Notwithstanding the above mentioned advantages of using cross-validated offsets, we nevertheless also include the version of priority-Lasso without cross-validated offsets in our application study (see “Results” section) for several reasons. Firstly, because the version with cross-validated offsets is more computationally intensive, and thus might not be easily applicable in all situations. Secondly, we aim to illustrate that this version tends to accredit more influence to the blocks with lower priority than does the version without cross-validated offsets. In addition, the suspected tendency of the version without cross-validated offsets to exclude blocks with lower priority might be advantageous in applications in which these blocks contain data types that are expensive to collect or not well established.

### R package prioritylasso

The priority-Lasso method (for continuous, binary, and survival outcomes) is implemented in the function ‘prioritylasso’ from our new R package of the same name (version 0.2), which is publicly available from the “Comprehensive R Archive Network” repository. This package uses the implementation of Lasso regression provided by the R package ‘glmnet’ (see [17], and for the special case of Cox-Lasso, see [18]).

The *M* penalty parameters $\lambda ^{(\pi _{1})},\dots,\lambda ^{(\pi _{M})}$ are chosen via cross-validation in the corresponding steps. As in ‘glmnet’, two variants are implemented: The penalty parameter can be chosen either in such a way that the mean cross-validated error is minimal (denoted as ‘lambda.min’), or in such a way that it yields the sparsest model with error within one standard error of the minimum (denoted as ‘lambda.1se’). The latter option yields sparser models. In order to further enforce sparsity at the convenience of the clinician, our package allows to specify a maximum number of non-zero coefficients for each block.

Furthermore, the function ‘prioritylasso’ offers the option to leave the block with highest priority unpenalized (i.e., to set $\phantom {\dot {i}\!}\lambda ^{(\pi _{1})}$ to 0), provided the number of variables $p_{\pi _{1}}$ in this group is smaller than the sample size *n*. Depending on the outcome, the estimation is then performed via generalized linear regression or via Cox regression [19]. Another variant of the priority-Lasso method is implemented in the function ‘cvm_prioritylasso’, which makes it possible to take more than one vector ***π*** as the input and choose the best one through minimizing the cross-validation error. This variant is useful in cases where it makes sense to take the group structure into account but the clinician does not feel comfortable assigning clear-cut priorities to each of the groups.

Note that our package solely aims at building prediction models with different types of already prepared omics data available as an *n*×*p* data matrix. However, generating such multi-omics data matrices from several types of raw data files requires considerable effort. We refer to Bioconductor software packages [20] that allow convenient annotation and organization of multi omics data. As an important example, the ‘MultiAssayExperiment’ data class [21] can be used for data preparation prior to running ‘prioritylasso’.

### Validation

In “Results” section, we apply the priority-Lasso method as well as the classical Lasso to fit prediction models for a time-to-event on a training dataset and subsequently evaluate these models on a validation dataset; see “AML data” section for a description of the data used in this analysis. The present section briefly describes the criteria considered to assess prediction accuracy and the procedures used for validation of the considered models, following the recommendations of Royston and Altman [14]. These authors emphasize in their paper that validation comprises both discrimination and calibration. Hence, we perform both in our analysis and focus on the methods denoted as methods 3, 4, 6, and 7 in their paper.

Firstly, following method 3, we present some measures of discrimination. Instead of Harrell’s C-index, a common measure to quantify the goodness of fit, we show the results of the Uno’s C-index [22], an adapted version of Harrell’s C-index that accounts for censored data and is thus more appropriate in our context. Another useful measure is the integrated Brier score [23] assessing both calibration and discrimination simultaneously, which we calculate over two different time spans: up to two years and up to the time of the last event. To visualize the results, we also show the corresponding prediction error curves obtained using the R package ‘pec’ [24].

Secondly, following method 4 of Royston and Altman [14], we display Kaplan-Meier curves that can be useful for both discrimination and calibration. For each considered prediction model, we define three risk groups, which corresponds to standard practice in the AML context. See for example the newest European Leukemia Net (ELN) genetic risk stratification of AML, which classifies patients into a low-, intermediate-, and a high-risk group [1] and will be referred to as ELN2017 score in the sequel. To build three groups based on a considered score, we choose the two cutpoints that yield the highest logrank statistic in the training data. We then present the Kaplan-Meier curves of the three risk groups for both training and validation sets. Good separation of the three curves in the validation dataset indicates good discrimination.

These three Kaplan-Meier curves observed for the validation dataset can also be compared to the predicted curves for the three risk groups in the validation dataset (Royston and Altman’s method 7). By “predicted curve for a risk group”, we mean the average of the individual predicted curves of the patients within this risk group. Good agreement between observed and predicted curves suggests good calibration. Thirdly, as an extension of the graphical check for discrimination, we also examine the hazard ratios across risk groups (Royston and Altman’s method 6).

Beyond these methods, we report the AUC, the true positive rate (TPR, also known as sensitivity) and the true negative rate (TNR, also known as specificity) of each score at two years after the diagnosis. This time point was chosen because its ratio of cases to survivors is the closest to 1. The true positive and the true negative rate are calculated with the median of each score as a cutoff for categorizing the scores into two groups. Furthermore, we consider a modified version of Royston and Altman’s method 1. They suggest performing a regression with the linear predictor from the model as the only covariate. For a standard Cox model the resulting coefficient is exactly 1 in the training data and should be approximately 1 in the validation data to indicate a good model fit. However, since we perform penalized regression this method is not applicable to our model. Therefore, we modify this criterion in calculating the calibration slopes in both training and validation data. The difference between the slope obtained using the training data and the one obtained using the validation data is a measure for the extent of the overoptimistic assessment of discrimination ability that is obtained using the training data.

## Results

The section starts with a brief description of the AML example dataset (“AML data” section). Then we present four models fitted using priority-Lasso (“Results of priority-Lasso” section) and compare them with the current clinical standard model and with two models fitted through standard Lasso (i.e., without taking the block structure into account) in terms of included variables (“Assessing included variables” section) and performance in the independent validation data (“Assessing prediction accuracy” section). These models are all fitted with a restricted number of selected variables. The same models without restrictions to the number of variables are presented in Additional file 1 for further comparisons. The complete R code written to perform the analyses is available from Additional file 2.

### AML data

In this study we use two independent datasets, denoted training set and validation set hereafter, including variables belonging to different blocks (see details below). All patients included in the analysis received cytarabine and anthracycline based induction treatment. The training set consists of 447 patients randomized and treated in the multicenter phase III AMLCG-1999 trial (clinicaltrials.gov identifier NCT00266136) between 1999 and 2005 [25, 26]. The patients are part of a previously published gene expression dataset (GSE37642) analyzed with Affymetrix arrays [27]. All patients with a t(15;17) or myelodysplastic syndrome are excluded, as well as patients with missing data.

The validation set consists of all patients with available material treated in the AMLCG-2008 study (NCT01382147) [28], a randomized, multicenter phase III trial (*n*=210) and additional *n*=40 patients that had resistant disease and were treated in the AMLCG-1999 trial. The dataset is publicly available at the Gene Expression Omnibus repository (GSE106291). The detailed inclusion and exclusion criteria were described previously [29]. The patients of the validation set were analyzed by RNAseq. For comparability, all continuous variables are standardized to a mean zero and variance one. All study protocols are in accordance with the Declaration of Helsinki and approved by the institutional review boards of the participating centers. All patients provided written informed consent for inclusion on the clinical trial and genetic analyses.

### Results of priority-Lasso

We apply priority-Lasso on the training dataset (*n*=447, described in “AML data” section), considering four different scenarios. These scenarios differ in the way the score ELN2017 is included in the analysis and whether or not the offsets are cross-validated (see “Formalization of priority-Lasso” section). Furthermore, we always apply the ‘lambda.min’ procedure and 10-fold-cross-validation for the choice of the penalty parameter in each step. However, since prediction performance is not the main concern in our analyses, the ‘lambda.1se’ approach would also be a reasonable option. In “Sensitivity analysis” section we show some results with ‘lambda.1se’ in addition to our main analyses. Furthermore, we allow for a maximum of 10 gene expression variables for each scenario as we want to keep the resulting model as simple as possible and experience has shown that in survival prediction for AML patients only a few gene expression values have a considerable influence on the outcome. Moreover, gene expression values are not easy to implement in clinical routine. We define the following blocks and corresponding priorities:

- Block of priority 1: the score ELN2017 [1]. It can be represented in different ways which are explained in the definition of the scenarios.
- Block of priority 2: 8 clinical variables measured at different scales
- Block of priority 3: 40 binary variables, each of which represents the mutation status for a certain gene
- Block of priority 4: 15809 continuous variables, each of which is the expression value of a certain gene

The order of these blocks have been determined by a physician involved in the project, who has many years of experience in the treatment of patients with AML, as well as experience with AML outcome prediction. These choices are based on practical considerations. However, alternative block orders could be reasonable from other points of view. For example, if the focus is solely on the maximization of prediction performance without any practical constraints, we refer to the function ‘cvm_prioritylasso’ from our R package ’prioritylasso’ which chooses the best order of blocks from two or more priority options according to the mean cross-validated performance. In addition to our main analyses that are based on an ordering that takes practical aspects into account as outlined above, we present additional results obtained for other block orders in “Sensitivity analysis” section.

#### Scenario pl1A

In the first scenario, the block of priority 1 consists of the three-categorical ELN2017 score represented by two dummy variables. We do not penalize this block and do not use cross-validated offsets. In this scenario the selected model includes only 7 variables represented by 8 coefficients: the dummy variables ELN2017_2 and ELN2017_3, equaling 1 for the intermediate and the high-risk category, respectively, and 0 otherwise, are selected by definition, because they result from a fit of a standard Cox model without penalization. Moreover, age, the Eastern Cooperative Oncology Group performance status (ECOG) [30], white blood cell count (WBC), lactate dehydrogenase serum level (LDH), hemoglobin level (Hb) and platelet count (PLT) are selected. The selected variables and their coefficients are displayed in the second and third column of Table 1. Variables from blocks with priority 3 (mutation status of 40 genes) and 4 (gene expression) are absent from the model, yielding a particularly sparse model based on variables which are easy to access.

**Table 1:** Variables selected by priority-Lasso in scenarios pl1A and pl1B

Block	Variable	Coef. pl1A	Coef. pl1B
1	ELN2017_2	0.8552	0.8552
	ELN2017_3	1.4324	1.4324
2	Age	0.3540	0.3556
	ECOG (> 1)	0.2794	0.2768
	WBC	0.1029	0.1019
	LDH	0.1744	0.1763
	Hb	0.0529	0.0532
	PLT	-0.0788	-0.0800
4	PHGDH		0.1242
	FAM171B		0.0726
	SH3PXD2B		0.0192
	F12		0.0097
	CD109		0.0599
	FAM92A1		0.0193
	LAPTM4B		0.0079
	FAM24B		0.0378
	DDIT4		0.0424
	DOCK1		0.0295

Column 1: priority of the block the variables are included in. Column 2: variable name. Column 3 and 4: coefficient of the variable in the Cox Lasso model

#### Scenario pl1B

This scenario is very similar to pl1A with the difference that the offsets are cross-validated as described in “Formalization of priority-Lasso” section. Because there are no offsets in the first step of the model fit, the coefficients of pl1A and pl1B are the same for the block of priority 1 (see Table 1, column 4). For the block of priority 2, the same variables are selected with small differences in their coefficients. While both models do not select variables from the block of priority 3, model pl1B additionally includes 10 gene expression markers—all with only small influence though. Nevertheless, the fact that gene expression markers are included in the model with cross-validated offsets, but not in the model without cross-validated offsets, illustrates the conjecture made in “Formalization of priority-Lasso” section: When using the priority-Lasso version with cross-validated offsets, more influence tends to be accredited to the blocks with lower priority compared to when using the version without cross-validated offsets.

#### Scenario pl2A

As an alternative approach, considered as sensitivity analysis in the present paper, one may also replace ELN2017 with the 19 variables that are used for its calculation. Because of the far higher number of variables, we penalize this block of priority 1. The results of the scenario without cross-validated offsets (scenario pl2A) are displayed in the third column of Table 2, showing that 14 of these 19 variables are selected. While the selected variables from block 2 are almost the same as in scenario pl1A (except the additional inclusion of sex), now there are 8 gene expression variables selected from the block of priority 4. We can see that these gene expression variables are not necessarily the same as in scenario pl1B.

**Table 2:** Variables selected by priority-Lasso in scenarios pl2A and pl2B

Block	Variable	Coef. pl2A	Coef. pl2B
1	t(8;21)(q22;q22)	-1.0289	-1.0289
	inv(16)(p13.1q22)	-1.5444	-1.5444
	NPM1 mut/FLT3-ITD neg or low	-1.0181	-1.0181
	biCEBPA	-1.2240	-1.2240
	NPM1 wt/FLT3-ITD pos or low	-0.4358	-0.4358
	t(9;11)(p21;q23)	0.4635	0.4635
	Other aberrations	-0.4376	-0.4376
	KMT2A rearrangements	-0.5440	-0.5440
	Complex karyotype	0.2970	0.2970
	Monosomal karyotype	0.0313	0.0313
	NPM1 wt/FLT3-ITD pos	0.1712	0.1712
	RUNX1 mutations	0.3065	0.3065
	ASXL mutations	-0.1224	-0.1224
	TP53 mutations	0.4306	0.4306
2	Age	0.2957	0.2617
	Sex	-0.1011	
	ECOG (> 1)	0.3147	0.3206
	WBC	0.0990	0.0589
	LDH	0.1681	0.2371
	Hb	0.0700	0.0671
	PLT	-0.0960	-0.0578
4	ZBTB37	0.0047	0.0025
	MFI2	0.0090	
	**SH3PXD2B**	0.0013	0.0418
	PDK3	-0.0187	
	**FAM24B**	0.0248	
	SIK3	-0.0063	
	OR7A17	0.0039	
	TBC1D17	-0.0172	
	**PHGDH**		0.0488
	**FAM171B**		0.0134
	FGD5		0.0359
	F12		0.0238
	IRX1		-0.0090
	FAM92A1		0.0239
	DDIT4		0.0769
	HSPA2		0.0169

Column 1: priority of the block the variable is included in. Column 2: variable name. Column 3 and 4: coefficient of the variable in the Cox Lasso model. Variables from the block of priority 4 also appearing in Table 1 are marked in bold

#### Scenario pl2B

Analogously to scenarios pl1A and pl1B, scenario pl2B is the same as pl2A, except that the offsets are calculated with cross-validation. Column 4 of Table 2 contains the results from this model, showing only small differences in the block of priority 2, but again large differences in the selected gene expression markers.

### Assessing included variables

For assessing the fitted models with respect to the selected variables, we consider as a reference two standard Lasso models fitted to the training data using the whole set of variables without taking any block structure into account. The two models differ in the way ELN2017 is treated. In the first Lasso model (variant ‘Lasso1’) it is considered as the score represented by two dummy variables. In the second Lasso model it is represented by the 19 variables which are used for its definition (variant ‘Lasso2’). In order to allow for a fair comparison, we again use the ‘lambda.min’ procedure and 10-fold-cross-validation to choose the penalty *λ*. Moreover, we allow the selection of a maximum number of variables equal to the number of all variables in blocks 1-3 for priority-Lasso plus 10. This corresponds to the fact that we did not restrict the number of variables of blocks 1-3 for priority-Lasso, but set the maximum number of gene expression variables to 10. The resulting models (not shown) clearly select more variables than the models obtained with priority-Lasso. Especially the number of gene expression variables is much higher (43 for Lasso1 and 52 for Lasso2), whereas only age for both models and ELN2017_3 for Lasso1 are selected variables from other types of data. Hence, priority-Lasso favors variables from blocks with high priority compared to standard Lasso and yields models that include considerably less variables.

### Assessing prediction accuracy

**Fig. 1:**
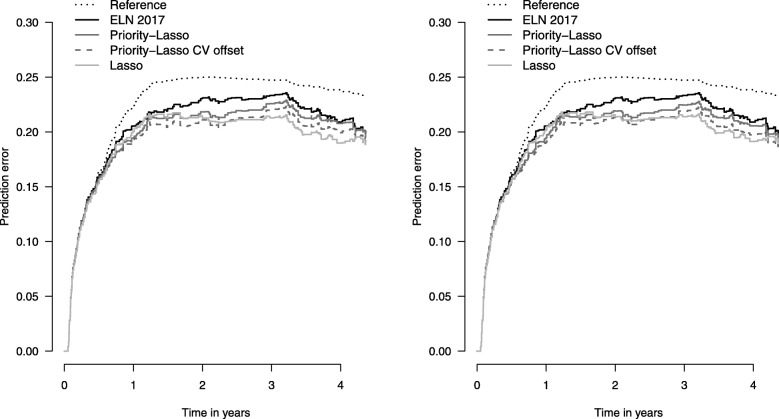
Prediction error curves. The curves show the Brier scores calculated in the validation data for the different scenarios and for different time points. The left panel contains the models considering ELN2017 as categories. The right panel contains the models considering all ELN variables. The Reference scenario results from the Kaplan-Meier estimation and is the same in both panels. Furthermore, curves for ELN2017, for priority-Lasso with and without cross-validated offsets, and for standard Lasso are shown

In order to compare the different approaches we follow the procedures described in “Validation” section − the results are shown in Table 3. It can be seen that pl1A and pl1B reach the highest sensitivity among the scenarios (0.672), whereas especially the raw ELN2017 score is associated with a far lower value (0.556). In contrast, the specificity is 0.723 for ELN2017, whereas all other scenarios are associated with a specificity between 0.64 and 0.67. However, these results represent only one of many possible time points and cutoffs, so their use is doubtful in our context. The other measures − the AUC, the C-indices, and the integrated Brier score − do not show great differences across the scenarios either. Only ELN2017 is an exception with considerably poorer results. For the AUC, pl1B yields the best result with a value of 0.731, but scenarios pl2B, Lasso1 and Lasso2 are not far worse. For C _*Uno*_, the highest value is 0.664, which is reached by pl2B. The integrated Brier score is calculated over two different time spans (up to 2 years and up to 4.4 years, the latter being the time to the last event). After two years, the priority-Lasso fit with cross-validated offsets is better than the other models − no matter how ELN2017 is treated. Over the whole time period, Lasso1 and pl2B give the lowest IBS, followed by Lasso2, indicating a lower prediction error for the Lasso models in the second half of the whole time period. This can also be observed in Fig. 1. Scenarios pl1B and pl2B perform best in the first two years but they are outperformed by Lasso afterwards. As expected, priority-Lasso with cross-validated offsets is always better than without. All fitted models are associated with a much lower prediction error than ELN2017 alone. The results from the prediction error curves do not differ substantially between the two panels of Fig. 1, that is, they are robust with regard to the handling of ELN2017.

**Table 3:** Validation results for the model scenarios with restrictions to the number of selected variables

	pl1A	pl1B	Lasso1	pl2A	pl2B	Lasso2	ELN2017
TPR	0.672	0.672	0.651	0.640	0.658	0.643	0.556
TNR	0.667	0.658	0.661	0.647	0.664	0.653	0.723
AUC	0.711	0.731	0.726	0.713	0.727	0.725	0.663
C _*Uno*_	0.653	0.660	0.658	0.658	0.664	0.656	0.619
IBS_2_	0.175	0.172	0.176	0.175	0.172	0.177	0.181
IBS _4.4_	0.197	0.192	0.191	0.197	0.191	0.193	0.204
Optimism	0.393	0.289	0.920	0.377	0.243	0.984	
CI$^{L}_{lower}$	0.339	0.304	0.247	0.387	0.327	0.177	0.418
HR ^*L*^	0.536	0.455	0.363	0.605	0.566	0.286	0.669
CI$^{L}_{upper}$	0.849	0.652	0.535	0.946	0.981	0.461	1.074
CI$^{H}_{lower}$	1.175	1.098	0.948	1.515	1.534	0.974	1.314
HR ^*H*^	1.751	1.651	1.385	2.208	2.199	1.386	1.954
CI$^{H}_{upper}$	2.612	2.483	2.022	3.216	3.151	1.972	2.907
*p*-value _*LR*_	1.11e-08	1.05e-8	2.22e-10	1.07e-08	1.74e-08	4.99e-11	1.36e-07

The acronyms in the first column are: TPR: True positive rate; TNR: True negative rate; AUC: Area under the curve, C _*Uno*_: Uno’s C-index, IBS_2_: Integrated Brier score up to 2 years, IBS _4.4_: Integrated Brier score up to 4.4 years, Optimism: difference between calibration slopes of training and validation data, CI$^{L}_{lower}$: lower bound of the 95% confidence interval for the hazard ratio of the low risk group, HR ^*L*^: hazard ratio of the low risk group, CI$^{L}_{upper}$: upper bound of the 95% confidence interval for the hazard ratio of the low risk group, CI$^{H}_{lower}$: lower bound of the 95% confidence interval for the hazard ratio of the high risk group, HR ^*H*^: hazard ratio of the high risk group, CI$^{H}_{upper}$: upper bound of the 95% confidence interval for the hazard ratio of the high risk group, *p*-value: *p*-value of the likelihood ratio test

**Fig. 2:**
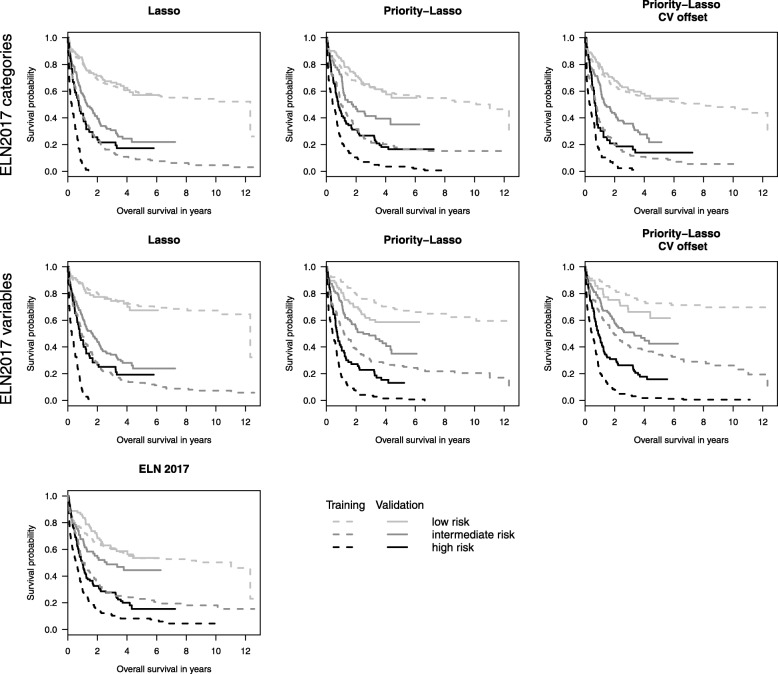
Kaplan-Meier curves for training and validation data in three risk groups. The three risk groups were built according to the highest logrank statistic in the training data. The left panel contains the results for the standard Lasso models and the raw ELN2017 score. The middle and right panels contain the plots of priority-Lasso with and without cross-validated offsets, respectively. The top and middle panels show the results considering ELN2017 as categories and using all ELN variables, respectively

The Kaplan-Meier curves for training and validation data are shown in Fig. 2. The discrimination by Lasso is obviously very good in the training data, but worse in the validation data. Especially the difference in survival between intermediate and high risk is not very clear. For both representations of ELN2017, the priority-Lasso models with and without cross-validated offsets feature a similar discrimination, where, however, the results obtained using the version with cross-validated offsets are slightly better. For the scenario with all ELN2017 variables, the priority-Lasso models give the best results in the validation data among all scenarios. In contrast, ELN2017 discriminates less well between the three risk groups. The results concerning Lasso indicate systematic overfitting in the training data. This is consistent with the results seen in “Assessing included variables” section where Lasso included much more variables than the other methods. It can also be seen from the row ‘optimism’ of Table 3. The difference of the slopes between training and validation data is the largest for the Lasso models, indicating that this method is associated with the highest overoptimism.

A possible way of quantifying the results seen in Fig. 2 is to consider the hazard ratios across risk groups in the validation set as shown in the lower half of Table 3. The intermediate group serves as a baseline here. The result of the likelihood ratio test is significant for all models. The discrimination between low and intermediate group is worst for the ELN2017 score. As already seen in Fig. 2, the discrimination between the low and intermediate group is better for Lasso than priority-Lasso. In contrast, priority-Lasso has a higher hazard ratio for the high risk group, in particular when using all ELN variables. These observations are also consistent with the results shown in Fig. 1, where the prediction was better for priority-Lasso than for Lasso in the earlier years, but worse in the later years. This corresponds to better prediction for shorter survival times and worse prediction for longer survival times, respectively. The fact that ELN2017 is included in the results of priority-Lasso, but not standard Lasso except ELN2017_3 in Lasso1, also seems to play a role for this issue. Both Fig. 2 and the hazard ratios clearly show that the prediction is better for high risk groups than for low risk groups with the raw ELN2017 score.

**Fig. 3:**
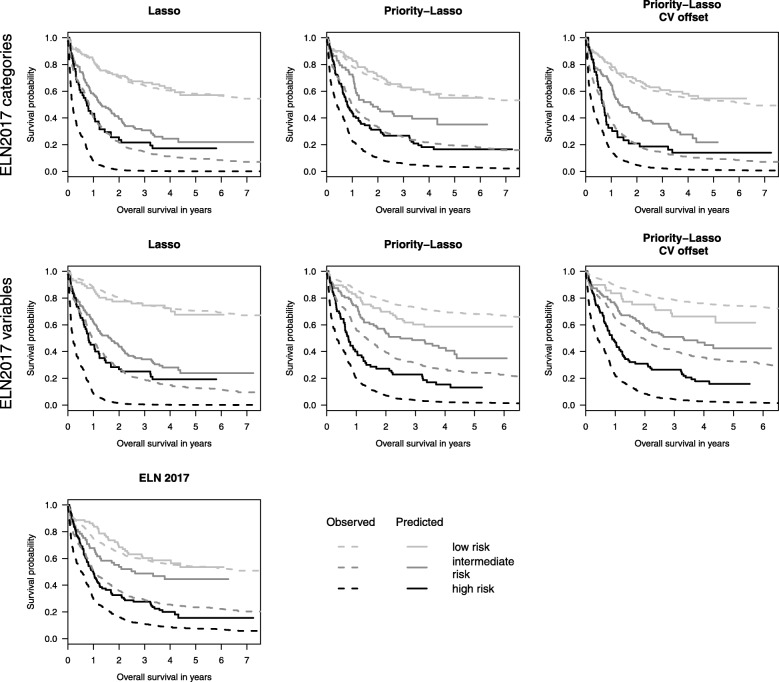
Observed and predicted Kaplan-Meier curves for the validation data in three risk groups. The three risk groups were built according to the highest logrank statistic in the training data. The left panel contains the results for the standard Lasso models and the raw ELN2017 score. The middle and right panels contain the plots of priority-Lasso with and without cross-validated offsets, respectively. The top and middle panels show the results considering ELN2017 as categories and using all ELN variables, respectively

Finally, we present the Kaplan-Meier curves for calibration in Fig. 3. For all the scenarios there are groups that reveal some miscalibration. For the Lasso models, especially the high risk groups differ between predicted and observed validation curves. The scenarios pl2A and pl2B show more differences between predictions and observations in the low risk groups than the other scenarios—the same fact applies to pl1A and pl1B in the intermediate risk group.

### Sensitivity analysis

In order to investigate the influence of different block orders on the selected variables, we run the four different scenarios of priority-Lasso with every possible block order (data not shown). The results show that the block order can have substantial influence on the number of selected variables. For the scenarios pl1A and pl1B, sparsest models are obtained with our priority definition, illustrating that priority-Lasso takes advantage of prior knowledge. Higher numbers of variables are obtained for other block orders with maximum values of 45 (pl2A, ***π***=(4,3,1,2) and ***π***=(4,3,2,1)). Seven of the eight selected variables in pl1A are chosen for almost every scenario of priority-Lasso and block orders, demonstrating their importance even in blocks of low priority. Remarkably, only a small part of them are found in the standard Lasso models (age in Lasso1 and Lasso2, as well as ELN2017_3 in Lasso1). It can be further observed that many of the selected gene expression variables are selected for only a small fraction of models.

In additional sensitivity analyses we consider the four scenarios with the ‘lambda.1se’ setting in order to choose the *M* values $\lambda ^{(\pi _{1})},\dots,\lambda ^{(\pi _{M})}$ as discussed in “R package prioritylasso” section. As expected, the ‘lambda.1se’ setting leads to a smaller number of selected variables for all scenarios. In total, the number of variables is 4, 10, and 15 for priority-Lasso with ELN categories, priority-Lasso with ELN variables (both with and without cross-validated offsets), and Lasso, respectively. The four different priority-Lasso models solely select variables from blocks 1 and 2. On the other hand, apart from age, Lasso selects only gene expression variables.

## Discussion

We introduced priority-Lasso, a simple Lasso-based intuitive procedure for patient outcome modelling based on blocks of multiple omics data that incorporates practical constraints and/or prior knowledge on the relevance of the blocks. The procedure essentially inherits most properties of Lasso. Its basic principle is however not limited to Lasso and could be easily adapted to recently developed variants of penalized regression.

An important feature of priority-Lasso is that it directly addresses the problem of redundancies in the predictive information across different blocks: Predictive information contained in the data from specific blocks is incorporated only if it is not contained in data from blocks of higher priority. To date, this idea seems to have been considered only in the TANDEM approach [31], that is, however, restricted to the case of two blocks.

In our illustrative example from leukemia research priority-Lasso was able to reach better prediction accuracy than Lasso. This applies especially to the version of priority-Lasso with cross-validated offsets, however, at the cost of more computation time and more selected variables than without cross-validated offsets. But even without cross-validated offsets, the models are not substantially worse than Lasso as far as accuracy is concerned. Moreover, they offer considerable advantages in terms of increased sparsity and composition of the models: they include less variables that are currently not included in the recommended diagnostic workup at initial diagnosis, which is an advantage from a practical perspective. Priority-Lasso offers more flexibility than Lasso: it allows the user to define block structures, where for each block a maximum number of selected variables can be specified.

The obtained models can be seen as compromises between “what the data tells us” and what is more realistic and easy to implement in clinical routine. As an extreme variant of priority-Lasso, one could imagine the case of a practitioner fixing the ordering of the variables completely, which amounts to considering blocks of size 1 (each variable forms one block). The other extreme consists of ignoring the block structure and simply fitting a model using Lasso to all variables. The finer the block structure, the less data-driven is the model selection. The number of blocks also influences the maximum possible number of selected variables in the final model. Since a maximum of *n* variables can be selected in a Lasso regression, a selection of *n* variables is the maximum for every block in priority-Lasso − hence the maximum possible number of variables selected by priority-Lasso depends on the number of blocks.

Unlike with Bayesian methods, prior knowledge is taken into account only through the definition and ordering of blocks. This feature makes the method less flexible, but also easy to use and interpret for scientists without strong background in statistics. The user does not have to perform any complicated choices in order to apply the method: The first choice to be made is whether or not the offset should be cross-validated — the variant without cross-validation gives more weight to blocks with high priority, but is prone to overfitting. Moreover, the user may decide to leave the block with highest priority unpenalized in case it satisfies $p_{\pi _{1}}< n$. By default it is treated like the other blocks of data and is thus penalized. As for all penalized regression methods, one can choose the procedure used for optimizing *λ* (in ‘glmnet’: *λ*_*min*_ or *λ*_1*se*_), which amounts to deciding between a more complex model with potentially slightly better accuracy and a sparser model. The default is *λ*_*min*_, that is, the *λ* associated with the minimum cross-validation error in each step. Of course there are additional parameters like the number of folds in the cross-validation procedures that could be modified as well, but are not expected to strongly affect the results.

Note that when working with multi-omics data other, more technical analysis steps are required before building prediction models. The package ‘prioritylasso’ itself was designed solely to build prediction models and takes the already formatted multi-omics data matrix as input. Fortunately, there are other tools available in Bioconductor that are of great value for the purpose of preparing multi-omics data. For example, the ‘MultiAssayExperiment’ software package [21] provides useful functions to represent, store, and operate on multi-omics data. It builds a bridge from standard R to Bioconductor and its classes for data representation that cannot be ignored in the context of omics data.

Finally, priority-Lasso offers further practical advantages for clinical practice. Suppose there are (blocks of) variables available only for a subset of patients and missing for the other. A potential approach to efficiently handle such data consists of assigning them a low priority in priority-Lasso. In this way, one can first fit a “basic” model to the blocks that are available for all patients, using all patients. This basic model can then be complemented by variables from the low priority blocks that are missing for a subset of the patients. Importantly, this is also relevant for prediction: Blocks which are not available for all patients in the training data will not be frequently available for new data for the purpose of prediction. In such cases, the basic prediction model can be used to obtain predictions.

## Conclusion

Our results show that priority-Lasso is a flexible and user-friendly prediction method that can reach a similar or even better prediction accuracy compared to standard Lasso. The feature which favors variables of blocks with higher priorities over variables of blocks with lower priority offers a practical advantage and makes the resulting prediction rules easy to use and interpret.

## Additional files

Additional file 1 Results of the analyses without restrictions to the maximum number of selected variables. (PDF 215 kb)

Additional file 2 R code written to perform the analyses. (ZIP 15 kb)

## Abbreviations

AML: Acute myeloid leukemia
AUC: Area under the curve
C-index: Concordance index
ECOG: Eastern cooperative oncology group
ELN: European leukemiaNet
Hb: Hemoglobin level
IBS: Integrated brier score
LDH: Lactate dehydrogenase serum level
PLT: Platelet count
RNAseq: Ribonucleic acid sequencing
TNR: True negative rate
TPR: True positive rate
WBC: White blood cell count
