# Supplementary material for: Priority-Lasso: a simple hierarchical approach to the prediction of clinical outcome using multi-omics data
Source: BMC Bioinformatics. 2018 Sep 12;19:322. doi: 10.1186/s12859-018-2344-6 (PMC6134797; doi:10.1186/s12859-018-2344-6)
Supplement: Supplementary file 1 — Results of the analyses without restrictions to the maximum number of selected variables. (PDF 215 kb) [file 12859_2018_2344_MOESM1_ESM.pdf]

Supporting information for the BMC Bioinformatics article:

Priority-Lasso: a simple hierarchical approach to the prediction of clinical outcome using multi-omics data

Simon Klau* Vindi Jurinovic Roman Hornung Tobias Herold
Anne-Laure Boulesteix

*Corresponding author: e-mail: simonklau@ibe.med.uni-muenchen.de, Department of Medical Information Processing, Biometry and Epidemiology, University of Munich, Marchioninistr. 15, D-81377, Munich, Germany

Results of the analyses without restrictions to the maximum number of selected variables

Supplementary Table 1: Number of variables selected for each model.

		Block 1	Block 2	Block 3	Block 4	Total
restricted	pl1A	2	6	0	0	8
	pl1B	2	6	0	10	18
	Lasso1	0	1	0	43	44
	pl2A	14	7	0	8	29
	pl2B	14	6	0	10	30
	Lasso2	0	1	0	52	53
not restricted	pl1A	2	6	0	0	8
	pl1B	2	6	0	35	43
	Lasso1	1	1	0	56	58
	pl2A	14	7	0	16	37
	pl2B	14	6	0	30	50
	Lasso2	0	1	0	57	58

Supplementary Table 2: Validation results for the model scenarios without restrictions to the number of selected variables. The acronyms in the first column are: TPR: True positive rate; TNR: True negative rate; AUC: Area under the curve, C_{Uno}: Uno's C-index, IBS_2: Integrated Brier score up to 2 years, $IBS_{4.4}$: Integrated Brier score up to 4.4 years, Optimism: difference between calibration slopes of training and validation data, CI^L_{lower}: lower bound of the 95% confidence interval for the hazard ratio of the low risk group, HR^L: hazard ratio of the low risk group, CI^L_{upper}: upper bound of the 95% confidence interval for the hazard ratio of the low risk group, CI^H_{lower}: lower bound of the 95% confidence interval for the hazard ratio of the high risk group, HR^H: hazard ratio of the high risk group, CI^H_{upper}: upper bound of the 95% confidence interval for the hazard ratio of the high risk group, p-value: p-value of the likelihood ratio test.

	pl1A	pl1B	Lasso1	pl2A	pl2B	Lasso2	ELN2017
TPR	0.672	0.655	0.669	0.640	0.666	0.666	0.556
TNR	0.667	0.650	0.672	0.647	0.672	0.678	0.723
AUC	0.711	0.744	0.734	0.716	0.741	0.722	0.663
C_{Uno}	0.653	0.671	0.667	0.659	0.669	0.656	0.619
IBS_2	0.175	0.170	0.178	0.175	0.169	0.178	0.181
$IBS_{4.4}$	0.197	0.188	0.193	0.196	0.187	0.193	0.204
Optimism	0.393	0.240	0.956	0.431	0.162	1.021	
CI^L_{lower}	0.339	0.238	0.166	0.316	0.335	0.190	0.418
HR^L	0.536	0.434	0.273	0.493	0.500	0.305	0.669
CI^L_{upper}	0.849	0.790	0.451	0.768	0.747	0.490	1.074
CI^H_{lower}	1.175	1.465	1.104	1.219	1.319	1.087	1.314
HR^H	1.751	2.103	1.529	1.800	2.018	1.538	1.954
CI^H_{upper}	2.612	3.020	2.231	2.657	3.088	2.176	2.907
p-value$_{LR}$	1.11e-08	1.98e-10	2.60e-12	2.32e-09	5.51e-10	1.76e-11	1.36e-07

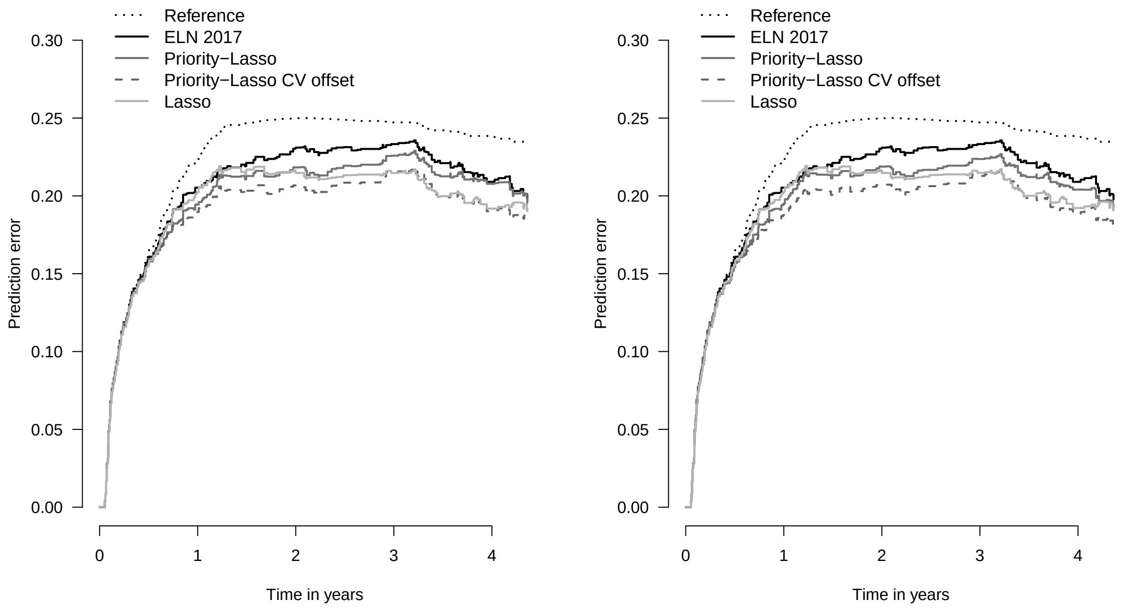

Supplementary Figure 1: Prediction error curves for models without restrictions to the maximum number of coefficients. The curves show the Brier scores calculated in the validation data for the different scenarios and for different time points. The left panel contains the models considering ELN2017 as categories. The right panel contains the models considering all ELN variables. The Reference scenario results from the Kaplan-Meier estimation and is the same in both panels. Furthermore, curves for ELN2017, for priority-Lasso with and without cross-validated offsets, and for standard Lasso are shown.

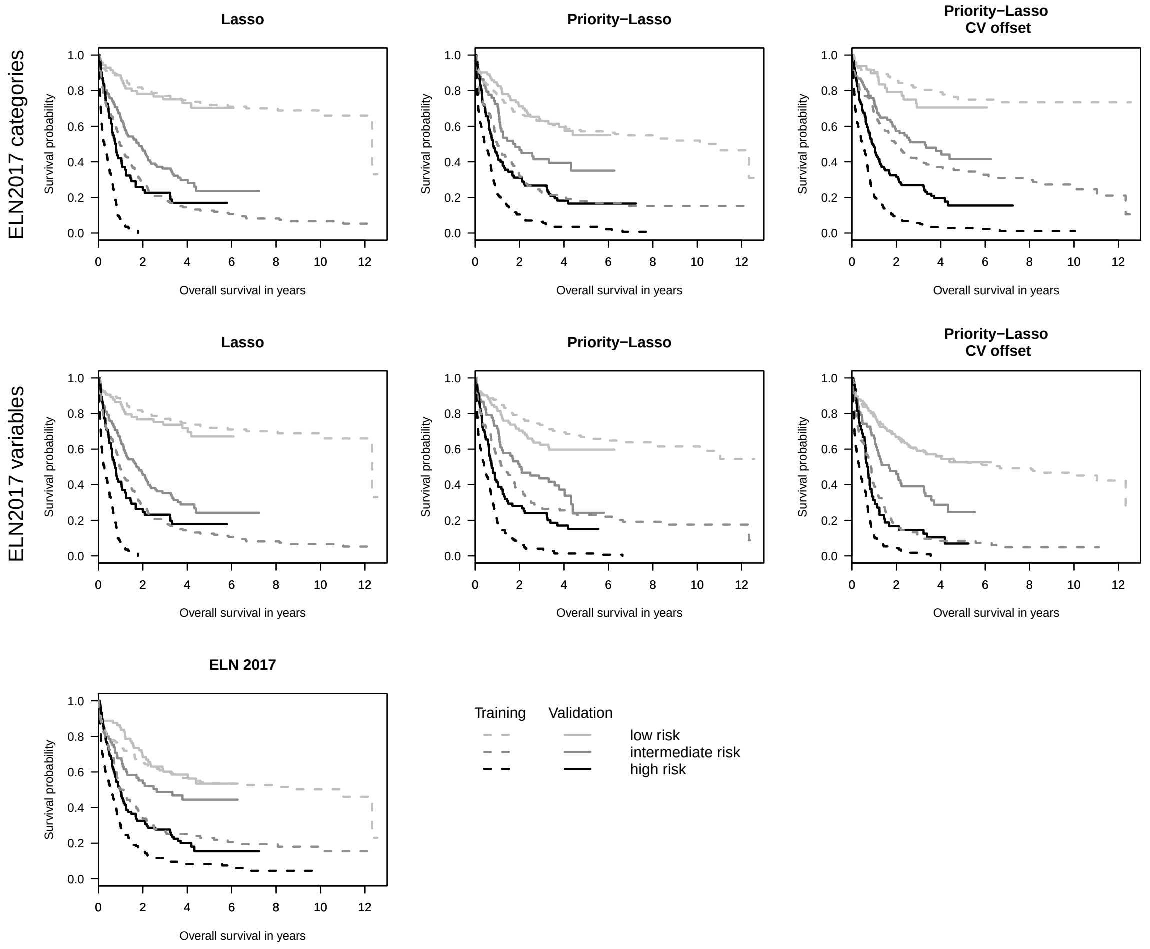

Supplementary Figure 2: Kaplan-Meier curves for training and validation data in three risk groups for models without restrictions to the maximum number of coefficients. The three risk groups were built according to the highest logrank statistic in the training data. The left panel contains the results for the standard Lasso models and the raw ELN2017 score. The middle and right panels contain the plots of priority-Lasso with and without cross-validated offsets, respectively. The top and middle panels show the results considering ELN2017 as categories and using all ELN variables, respectively.

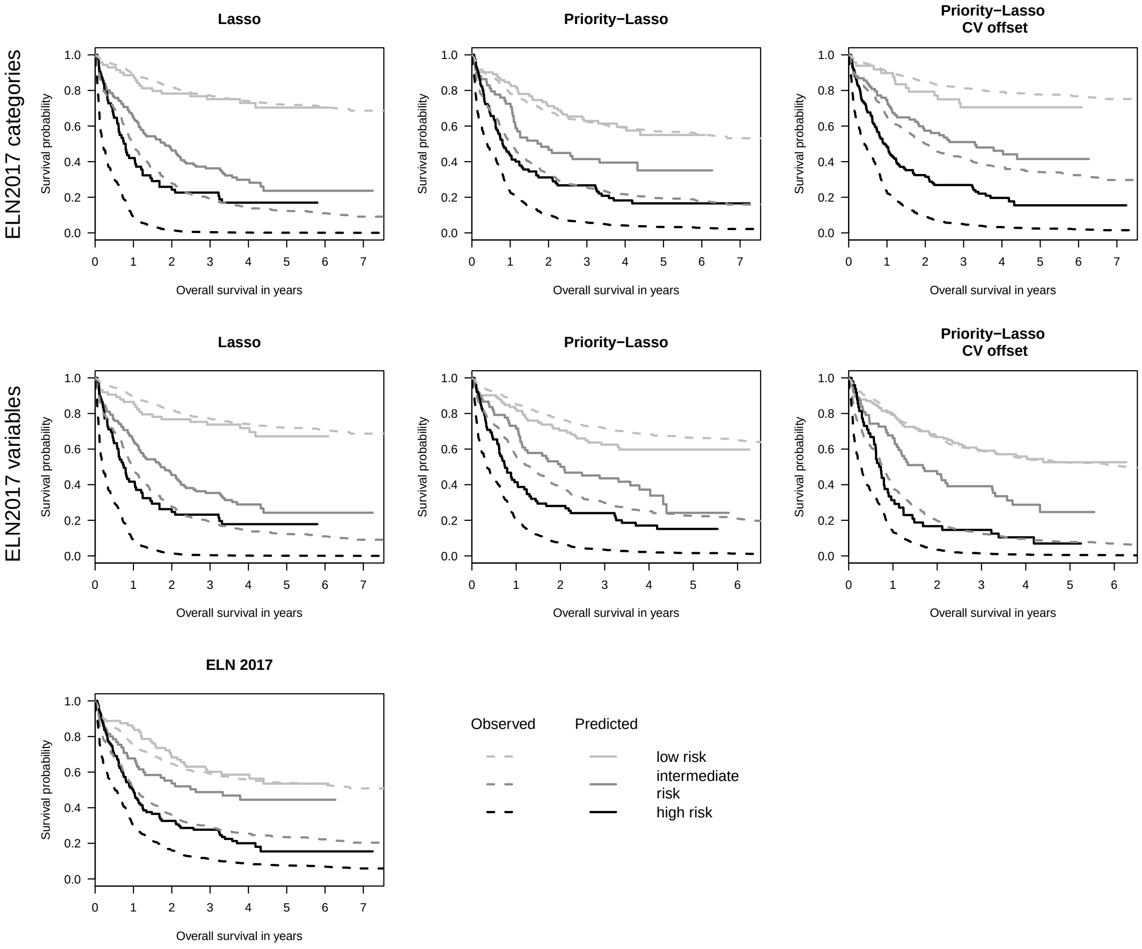

Supplementary Figure 3: Observed and predicted Kaplan-Meier curves for the validation data in three risk groups for models without restrictions to the maximum number of coefficients. The three risk groups were built according to the highest logrank statistic in the training data. The left panel contains the results for the standard Lasso models and the raw ELN2017 score. The middle and right panels contain the plots of priority-Lasso with and without cross-validated offsets, respectively. The top and middle panels show the results considering ELN2017 as categories and using all ELN variables, respectively.
